# Women’s Empowerment and Infant and Child Health Status in Sub-Saharan Africa: A Systematic Review

**DOI:** 10.1007/s10995-020-03025-y

**Published:** 2020-11-23

**Authors:** Solomon Kibret Abreha, Yacob Abrehe Zereyesus

**Affiliations:** 1 Department of Economics, University of Insubria, Varese, Italy; 2 Economic Research Service, U.S. Department of Agriculture, Kansas City, MO, USA

**Keywords:** Child health, Women's empowerment, Decision making, Anthropometric indicators, Sub-Saharan Africa

## Abstract

**Introduction:**

Although many studies have examined the relationship between women’s empowerment and a wide range of health outcomes, the extent to which the different dimensions of empowerment influence children’s health, and through which mechanisms and in what contexts, is limited in sub-Saharan Africa. The objective of this review is to systematically assess and examine studies that investigated the association between women’s empowerment and children’s health status in sub-Saharan Africa.

**Methods:**

A systematic review of the published literature is searched through PubMed, Google Scholar, Embase, Web of Science and Scopus databases focusing on different measures of women’s empowerment and children’s health outcomes. Inclusion criteria in the review are studies that are published in English; full and original articles; studies measuring at least one dimension of women’s empowerment and children’s health outcomes; and Sub-Saharan African context. Studies included in this review are articles published between the year 2000 and 2019. Studies were excluded if the source was a letter, editorial, review, commentary, abstracts without providing full information about the study.

**Results:**

Initially 4718 citations were identified. Finally, 15 studies met the inclusion and exclusion criteria. In general, the evidence suggests that women’s empowerment at the household level is positively and statistically significantly associated with better children’s health outcomes in sub-Saharan African countries. The review also reveals that women’s decision-making power or autonomy is the most common measure of women’s empowerment employed by many studies.

**Conclusions:**

Future related studies would benefit by incorporating additional aspects of women's empowerment and child health outcomes.

**Electronic supplementary material:**

The online version of this article (10.1007/s10995-020-03025-y) contains supplementary material, which is available to authorized users.

## Significance Statement

Many studies have examined the relationship between women’s empowerment and a wide range of health outcomes. However, the extent to which the different dimensions of empowerment influence children’s nutrition and health, and through which mechanisms and in what contexts, is limited in sub-Saharan Africa. This paper aims to systematically assess and examine studies that investigated the association between women’s empowerment and infant and children’s health status in sub-Saharan Africa. The review contributes to the existing literature highlighting the impact of women's empowerment on children's health outcomes in sub-Saharan African countries.

## Background

Women’s empowerment has received an increasing amount of attention over the last three decades among researchers and development organizations (Ballon 2018; Kabeer 1999). The United Nations (1995), the UNFPA (1994) and the UN General Assembly (2000) laid a foundation for the promotion of gender equality and women’s empowerment as a key strategy for development and policy goals (Ballon 2018; Bhattacharya and Banerjee 2012). Women’s empowerment and gender equality are development objectives in their own right, as embodied in Millennium Development Goals 3 and 5 (Razavi 2012; UN 2015) and as the 5th of 17 Sustainable Development Goals (United Nations General Assembly 2015). Kabeer (1999) defines women’s empowerment as "the process by which those who have been denied the ability to make strategic life choices acquire such an ability”. Kabeer (1999) considers women’s empowerment as the ability to exercise choice and intrinsic human right goal. Although some studies have focused on measuring women’s empowerment (Ewerling et al. 2017; Miedema et al. 2018), the association between women’s empowerment and children’s health status in developing countries in general and Sub-Saharan African countries, in particular, is limited.

Infant and child health has gained international development agenda over the last 20 years. For instance, the Millennium Development Goals (MDGs) and Sustainable Development Goals (SDGs) by the United Nations in 2000 and 2015, respectively have been dedicated to promoting healthy lives and well-being for all children, particularly MDG-4 which aims to reduce child mortality and SDG-3 which aims to end preventable deaths of newborns and children under 5 years by 2030 (UN 2015; UNDP 2015). Although progress had been made in reducing child mortality, an estimated 6.3 million children under the age of 15 years died in 2017 (IGME 2018). About 5.4 million of them were under the age of 5 and 2.5 millions of those children died within the 1st month of their life, equivalent to 15,000 under—5 deaths per day (IGME 2018).

In 2017, half of all deaths under 5 years of age took place in Sub-Saharan Africa (SSA) making the region with the highest under-5 mortality rate in the world. In SSA 1 out of 13 dies before his or her 5th birthday, 14 times higher than in high-income countries, wherein high-income countries, that number was 1 in 185 (IGME 2018). Childhood stunting and wasting are the most serious health problems in sub-Saharan African countries. Half of all deaths in children under 5 years are attributed to undernutrition. Stunting affected nearly two out of five children in sub-Saharan Africa in 2018 (UNICEF/WHO/World Bank 2019). The UNICEF/WHO/World Bank Joint Child Malnutrition Estimates (2019) report showed that in 2018 around the world, over 49 million children under five were wasted of which nearly 17 million were severely wasted (UNICEF/WHO/World Bank 2019). Hence, the prevalence rate of wasting is 7.3% and that of severely wasted is 2.4% (UNICEF/WHO/World Bank 2019). From this figure, 25% of wasted children lived in sub-Saharan Africa.

Women’s empowerment is critical to achieving development objectives and contributes to better family health outcomes including child health (Duflo 2012; Mehra 1997; Miedema et al. 2018; Pratley 2016). Women’s better access to health, education, and employment, increased political participation, control of resources including house and land are crucial for promoting sustainable development and improving health outcomes (PMNCH 2013). Women’s empowerment can lead to improvements in health and quality of life for women and their family members through many pathways. Women with greater empowerment are more likely to have fewer children, more likely to access health services and have control over health resources, and less likely to suffer domestic violence (Mabsout 2011; Pennington et al. 2018; Pratley 2016; Richards et al. 2013; Stiyaningsih and Wicaksono 2017). Their children are more likely to survive, receive better childcare at home and receive health care when they need it (Mabsout 2011; Pennington et al. 2018; Pratley 2016; Richards et al. 2013; Stiyaningsih and Wicaksono 2017).

Women’s empowerment is multidimensional and measured using multiple indicators (Kabeer 1999; Mabsout 2011; Mason 1986; Pratley 2016; Richardson 2018). Kabeer’s dimensions of women’s empowerment include resources (preconditions), agency (autonomy) and achievements (outcomes) (Kabeer 1999). According to Kabeer, resources and achievement are indirect measures of women’s empowerment while decision making is a direct measure of women’s empowerment. Using the 2011 Ethiopian Demographic and Health Survey (DHS) data, Mabsout (2011) measured women’s empowerment in Ethiopia using women's participation in decision making, women's earning, and years of education as major components of empowerment. A review by Pratley (2016) identified five broad dimensions of women’s empowerment measure to study the association between women’s empowerment and child health in developing countries. The dimensions are psychological, social, economic, legal and political. The variables included to measure the economic and social dimensions of empowerment are health indicators such as decision making on own healthcare, where to take children in case of illness, freedom to visit a doctor and exposure to intimate partner violence (Pratley 2016). Zereyesus et al. (2017) used the recently developed Women’s Empowerment in Agriculture Index (WEAI) that is composed of five domains of empowerment and its association with children’s health status in northern Ghana. Deutsch and Silber (2017) also studied women’s empowerment in Mozambique by including women’s participation in decision-making, actual use of violence by husband, attitude towards the use of violence, resources of household and information as the major dimensions of women’s empowerment. Ndaimani et al. (2018) used decision-making (visiting relatives and friends, healthcare, household purchases, husband earnings) and ownership of assets (house ownership, land ownership, title deed ownership, and land title deed ownership) to measure the association between women’s empowerment and uptake of child health services using the 2016 Zimbabwe Demographic and Health Survey. However, differences in indicators to measure empowerment between studies and other context-specific factors make it difficult to compare between studies and to interpret the results (Cunningham et al. 2015; Pratley 2016). To address this gap, this systematic review observes how different studies defined and measured women’s empowerment and how the different dimensions of women’s empowerment are associated with child health indicators in sub-Saharan African countries.

Understanding the major causes of death among children and consolidating the existing evidence regarding women’s empowerment as a determinant in children’s health is an essential step towards improving children’s health and particularly so in sub-Saharan African region which happens to shoulder a more than proportionate share of the child-related health burden in the world. This systematic review aims to generate a synthesis of the existing evidence regarding the association of women’s empowerment and children’s health and nutritional status in sub-Saharan Africa and provides recommendations for future research.

## Methods

### Search Strategy

A systematic review of the published literature was conducted to identify and examine previous studies that investigated the associations between women’s empowerment and children’s health status in sub-Saharan African countries. Using key search terms incorporating the words infant, child, health, women, and empowerment, [see Table 1] and a search strategy as provided in Appendix A of the additional file, the relevant literature was searched through PubMed, Google Scholar, Embase, Web of Science, Scopus and SSRN electronic library. The search strategy is aimed at incorporating academic literature. The documents were searched between May and June 2019. The search generated 4718 studies for abstract and title screening. The results are reported in line with the PRISMA guidelines for reporting systematic reviews (Moher et al. 2009).

**Table 1:** Search Terms

Subject	Components of women’s empowerment	Search terms	Children’s health indicators	Locations
Women’s OR Female OR Mother OR Maternal AND Child OR Children OR Infant	Women’s empowerment OR Decision making OR Women’s autonomy OR Women’s agency OR Women’s control OR	AND	Child health status OR Child health outcome OR Anemia Pneumonia Child stunting Child wasting Underweight	Sub-Saharan African countries

### Inclusion and Exclusion Criteria

The review includes studies written in the English language and published in the period from 2000 to 2019 that examined the relationship(s) of at least one women’s empowerment domain and children’s health outcomes in Sub-Saharan African countries. The year 2000 was chosen as the cut-off for the systematic review because the nationally representative Demographic and Health Survey (DHS) data funded by the U.S. Agency for International Development (USAID), in which most studies on women’s empowerment in Sub-Saharan Africa are based, started to rigorously incorporate variables that capture women’s empowerment in this particular year. Study subjects include women and children and target studies include full articles focused on quantitative methods. The review includes studies measuring at least one dimension of women’s empowerment and its constituent indicators. The components of women’s empowerment include decision making, autonomy, agency, socioeconomic status, attitudes towards violence and asset ownership. The outcomes are different indicators of child health such as infant mortality, stunting, wasting, underweight, immunization, anemia and pneumonia indicators. Studies were excluded if the source was a letter, editorial, review, commentary, abstracts without providing full information about the study. We specify the patient intervention comparator outcome (PICO) strategy in the additional material Appendix B.

## Results

### Descriptive Characteristics of Included Studies

**Fig. 1:**
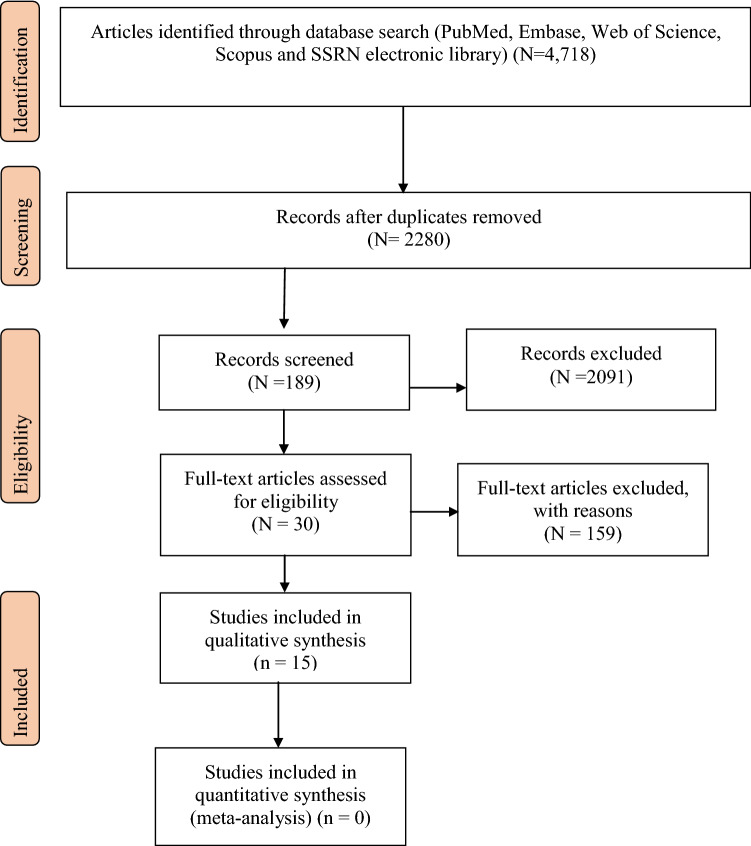
Preferred reporting items for systematic reviews (Moher et al. 2009)

Figure 1 presents the preferred reporting item for the diagram of the systematic review. The search process identified 4718 articles and reports published in the period between 2000 and 2019. After removing 2438 duplicates, 2280 articles were screened for eligibility. Another 2091 records were further excluded because they did not meet the study location and the topic criteria. Additionally, 159 full articles were excluded because they do not report empowerment dimensions, child health outcomes. The remaining 30 full-text articles were further assessed for eligibility. These studies were critically appraised considering the appropriateness of study design to the research questions, statistical issues and risk of bias (Tacconelli 2010). Based on this, studies that meet a set of pre-defined minimum quality criteria in terms of study design and data collection, confounding, selection bias and statistical issues were included in the review. Based on these criteria, each of the studies was assessed and given a score for quality rating. Of these 15 studies that demonstrated higher quality (rating “A” or “B”) were selected for the final review and the remaining 15 studies that scored “C” were excluded from the review. Finally, 15 studies that satisfied the inclusion and exclusion were selected for the final review. For more, see Appendix C of the additional material.

Table 2 presents the characteristics of the included studies. The studies published in the period from 2005 to 2019. The majority of studies used secondary data analysis and cross-sectional surveys such as the Demographic and Health Survey (DHS) (Alemayehu et al. 2015; Deutsch and Silber 2017; Ibrahim et al. 2015; Jones et al. 2019; Ndaimani et al. 2018; Ross-Suits 2010). Only one study used a randomized control trial design to study the association of women’s empowerment and child’s nutritional outcome (Heckert et al. 2019). Heckert et al. (2019) studied the associations between women’s empowerment and child nutritional status using data from a cluster randomized control trial. The studies were mostly focused in Nigeria (Akinyemi et al. 2017; Ibrahim et al. 2015); Ethiopia (Jones et al. 2019; Alemayehu et al. 2015; Fantahun et al. 2007), and Ghana (Zereyesus et al. 2017; Malapit and Quisumbing 2015). Three studies are multi-country studies that covered several sub-Saharan African countries (Desai and Johnson 2005; Jones et al. 2019; Na et al. 2015).The majority of the studies applied a multivariate regression method to analyze the associations of women’s empowerment and children’s health (Alaofè et al. 2017; Alemayehu et al. 2015; Ibrahim et al. 2015; Na et al. 2015; Ndaimani et al. 2018). Three studies applied structural equation modeling and Multiple Indicators Multiple Causes (MIMIC) models (Deutsch and Silber 2017; Zereyesus et al. 2017; Jones et al. 2019). Two used hierarchical linear models to distinguish between individual and community level influences of women empowerment (Brunson et al. 2009; Desai and Johnson 2005).

**Table 2:** Characteristics of included studies

(Author, year)	Data source/country	Design/Method	Confounders (control variables)
(Heckert et al. 2019)	Burkina Faso	Cluster-randomized controlled trial/structural equation models	Child’s gender, child’s age, maternal age, maternal height
(Jones et al. 2019)	Demographic and Health Surveys (2011–2016) Ethiopia, Kenya, Rwanda, Tanzania, and Uganda	Cross-sectional/structural equation modeling	Women’s age, education, parity, Women’s BMI, household wealth, urban, male child
(Ndaimani et al. 2018)	2016 DHS/Zimbabwe	Cross-sectional/mixed methods (pairwise correlation, multivariate regression, adjusted logistic regressions)	Women’s education, Women’s age, Spousal age difference, marital status, occupation Type of health insurance, Place of residence Locality, Number of children Children’s age, sex, birth order
(Zereyesus et al. 2017)	2012 population-based survey data of the Feed the Future food security program/Ghana	Cross-sectional/Multiple Indicators Multiple Causes (MIMIC) model	Child's age, Child's gender, women's education women's age, women's dietary diversity score Father's education, Household hunger scale Income deciles, Household size, Safe drinking water
(Deutsch and Silber 2017)	2009 DHS/Mozambique	Cross-sectional/MIMIC weighting (fuzzy approach)	Characteristics of women: (education, age, BMI, gender) Regional variables Female child Region dummies (rural/urban)
(Alaofè et al. 2017)	2014 Solar Market Garden baseline study/Northern Benin	Cross-sectional/exploratory and confirmatory factor analysis Univariate analysis Multiple regression analysis	Maternal characteristics (Age in years, level of education completed, occupation, dietary diversity score), Child characteristics (age in months and sex), and Household characteristics (Household size, Religion, Ethnicity)
(Akinyemi et al. 2017)	2013 DHS Nigeria	Cross-sectional Kaplan–Meier estimates of childhood mortality Multilevel discrete-time hazard models	Maternal age at child's birth (years), Maternal education, Household wealth index, Maternal religion, Birth order, Preceding birth interval
(Ibrahim et al. 2015)	2008 DHS/Nigeria	Cross-sectional/Chi-square test Logistic regression	Women’s age, women’s education, women’s occupation, Parity, Child’s age Father’s age, father’s education, Place of residence, wealth status
(Alemayehu et al. 2015)	2000, 2005, 2011 DHS/Ethiopia	Secondary serial cross-sectional analysis, univariate, bivariate, and multivariate analyses	Women’s educational status Age in years, type of residential area, husband’s education, wealth index
(Na et al. 2015)	sub-Saharan Countries 2010 and 2013 DHS	Cross-sectional Multivariate logistic regression	Maternal age, maternal education, child age, child sex, number of living children, residence, household wealth quintile
(Malapit and Quisumbing 2015)	2012 population-based survey data of the Feed the Future food security program/Ghana	Cross-sectional Probit regression	Child’s age and sex, women’s age, women’s height, women’s education
(Ross-Suits 2010)	Tanzania 2004–2005 DHS	Cross-sectional Logistic regression	Child age, child sex, birth order, duration of breastfeeding, place of residence, maternal education, maternal employment, religion, family wealth index, family size
(Brunson et al. 2009)	Kenya Data collected from residents	Cross-sectional survey Analysis of variance Hierarchical linear modeling (HLM|)	Maternal education, Economic status maternal age, household size, marriage type
(Fantahun et al. 2007)	Data collected from Butajira Demographic Surveillance Site Ethiopia	Prospective case–control a multivariate model for conditional logistic regression	Reproductive patterns, woman’s age, Number of pregnancies, Child Care, Health care-seeking
(Desai and Johnson 2005)	DHS/cross-country (Benin, Malawi, Mali, Uganda, and Zimbabwe in sub-Saharan Africa)	Hierarchical linear models	women's education (categories) her partner's education household wealth index the historical period of birth urban–rural

The studies used different individual, household and community-level variables known from the literature to correlate with women’s empowerment and children’s health outcomes. The variables mainly classified into individual-level factors which include women’s education, women’s age, occupation, child’s age and sex, father’s education (Alaofè et al. 2017; Alemayehu et al. 2015; Heckert et al. 2019; Zereyesus et al. 2017), household-level characteristics (household size, income, household hunger scale (Alaofè et al. 2017; Ibrahim et al. 2015; Na et al. 2015; Zereyesus et al. 2017) and community-level characteristics of residence and region. Although women’s education level is considered as an enabling factor or indirect measure of women’s empowerment in Kabeer’s framework, most of the studies included in this systematic review considered it as a control variable together with other individual socio-economic variables. Only one study acknowledges women’s education as an enabling factor of women’s empowerment but it is excluded from the analysis due to inconsistent and low factor loadings across countries included in the analysis (Jones et al. 2019). In other studies, education is the most commonly used indicator of women’s empowerment (Asaolu et al. 2018; Mabsout 2011; Pratley 2016).

### Women’s Empowerment Dimensions

The studies used various definitions and measurements of women’s empowerment. The most common definition of empowerment is “ the process by which those who have been denied the ability to make strategic life choices acquire such an ability (Kabeer 1999). In addition to this, some authors defined it as “the process of women’s enhancement of their ability to make strategic life choices” (Ndaimani et al. 2018; Zereyesus et al. 2017). In general, in this systematic review, we identified and categorized five major domains of women’s empowerment (See Table 3). The description of each of the five domains of women’s empowerment is presented below.

**Table 3:** Women empowerment and child health outcomes considered in the studies

Variables	Studies
*Women empowerment domains*	
Decision making, Women’s autonomy or agency	(Akinyemi et al. 2017; Alaofè et al. 2017; Alemayehu et al. 2015; Brunson et al. 2009; Deutsch and Silber 2017; Fantahun et al. 2007; Heckert et al. 2019; Ibrahim et al. 2015; Malapit and Quisumbing 2015; Na et al. 2015; Ndaimani et al. 2018; Ross-Suits 2010; Zereyesus et al. 2017; Jones et al. 2019)
Ownership of assets (house and land)	(Deutsch and Silber 2017; Na et al. 2015; Ndaimani et al. 2018; Zereyesus et al. 2017)
Domestic violence	(Deutsch and Silber 2017; Jones et al. 2019)
Mobility	(Alaofè et al. 2017)
Leadership	(Alaofè et al. 2017)
*Child health outcomes*	
Treatment of diarrhea	(Ndaimani et al. 2018)
Use of vaccinations	(Desai and Johnson 2005; Ibrahim et al. 2015; Ndaimani et al. 2018)
Anthropometric indicators (height-for-age and weight-for-height)	(Alaofè et al. 2017; Brunson et al. 2009; Desai and Johnson 2005; Deutsch and Silber 2017; Ibrahim et al. 2015; Malapit and Quisumbing 2015; Na et al. 2015; Ross-Suits 2010; Zereyesus et al. 2017; Jones et al. 2019)
Infant mortality	(Akinyemi et al. 2017; Alemayehu et al. 2015; Desai and Johnson 2005; Fantahun et al. 2007)

#### Decision Making

Decision making is the most common measure of women’s empowerment adopted by the majority of the studies (Heckert et al. 2019; Ndaimani et al. 2018; Zereyesus et al. 2017; Deutsch and Silber 2017; Alaofè et al. 2017; Akinyemi et al. 2017; Ibrahim et al. 2015; Alemayehu et al. 2015; Malapit and Quisumbing 2015; Na et al. 2015; Ross-Suits 2010; Brunson et al. 2009; Fantahun et al. 2007; Jones et al. 2019). Out of 15 studies included in this systematic review, 14 studies used decision making variables in one form or another. The studies used different terms to describe decision making variables such as women’s autonomy or agency. The most common decision-making indicators are indicators which taken from the Demographic and Health Survey. Hence, women’s participation in decision-making includes indicators such as decisions taken on the woman’s own healthcare; decisions on major household purchases; decisions related to visits to the woman’s family or relatives and decisions connected to control over woman’s earning.

#### Asset Ownership

Asset ownership is the second most used domain of women’s empowerment employed by the studies (Ndaimani et al. 2018; Zereyesus et al. 2017; Deutsch and Silber 2017; Na et al. 2015). This domain of empowerment includes indicators of women’s ownership of house and land, title deed ownership, land title deed ownership). This means an indicator for ownership of assets will be generated by summing land ownership, house ownership, land title deed ownership, and house title deed ownership (Ndaimani et al.^2018^).

#### Domestic Violence

This domain consider indicators such as wife-beating by the husband or partner for reasons like (a) burning food, (b) arguing with him, (c) going out without telling him, (d) neglecting the children, and (e) refusing to have sex with him. Hence, the dimension of women’s empowerment regarding domestic violence is constructed by incorporating these indicators regarding attitudes towards wife-beating (Jones et al. 2019).

#### Mobility

Mobility domain is characterized as women’s freedom to leave the home which includes indicators such as permission to go to market, permission to go to the health center or traditional doctor and permissions to go visit friends (Alaofè et al. 2017).

#### Leadership

Alaofè et al. (2017) considered the leadership domain as assertiveness and actions indicating a sense of self-security. The domain incorporates two indicators concerning the women’s sense of self-whether women feels important or her opinion was respected and women’s. self-confidence-whether she feels confident to resolve a problem on her own (Alaofè et al. 2017) The study by Zereyesus et al. (2017) and Malapit and Quisumbing (2015) also incorporated leadership (e.g. group membership and speaking in public) as a domain in women’s empowerment.

### Health Outcome Measures

The majority of the studies use children’s anthropometric indicators (height-for-age, weight-for-height, and weight-for-age) to represent children’s health outcomes. As indicated in Table 3 below, 11 of the 15 studies included in the systematic review employ children’s nutritional indicators to measure child health. Besides, four studies used child mortality measure (Akinyemi et al. 2017; Alemayehu et al. 2015; Desai and Johnson 2005; Fantahun et al. 2007). Ndaimani et al. (2018) used the prevalence of diarrhea to indicate child health outcomes. See Table 3 for more indicators of child health with their respective studies.

### Associations Between Women’s Empowerment and Children’s Health Outcomes in Sub-Saharan Africa

The empirical evidence regarding the relationship between women's empowerment and children’s health is diverse. In this review, we organized the evidences into two categories based on the health outcomes considered by the studies included in the systematic review. The first category of the evidence is children’s anthropometric indicators (height-for-age z-score, weight-for-height z-score and weight-for-age z-score) (Alaofè et al. 2017; Brunson et al. 2009; Desai and Johnson 2005; Deutsch and Silber 2017; Ewerling et al. 2017; Ibrahim et al. 2015; Malapit and Quisumbing 2015; Na et al. 2015; Ross-Suits 2010; Zereyesus et al. 2017; Jones et al. 2019).These anthropometric indicators are better measures of child health as they measure the nutritional status of infants and children using nutritional indices (WHO-CGS 2006). The second category of the evidence regarding relationship between women’s empowerment and children’s health outcome is related to children’s non-anthropometric health indicators. Such indicators include infant mortality, treatment of diarrhea and use of vaccinations (Ndaimani et al. 2018; Akinyemi et al. 2017; Alemayehu et al. 2015; Ibrahim et al. 2015; Fantahun et al. 2007; Desai and Johnson 2005). Below we discuss the results of the studies included in this systematic review regarding children’s anthropometric and non-anthropometric health indicators.

#### Women’s Empowerment and Children’s Anthropometric Health Indicators

The majority of the studies reported a consistent or positive association between women’s empowerment and anthropometric child health indicators. Of the 15 studies included in this review, the majority (53%) documented consistent positive relationships between different dimensions of women’s empowerment and anthropometric child health outcomes (Akinyemi et al. 2017; Alaofè et al. 2017; Alemayehu et al. 2015; Ewerling et al. 2017; Fantahun et al. 2007; Heckert et al. 2019; Ibrahim et al. 2015; Malapit and Quisumbing 2015).

Using Demographic and Health Surveys (2011–2016) data in five African countries (Ethiopia, Kenya, Rwanda, Tanzania, and Uganda), the study by Jones et al. (2019) examined the pathways by which women’s empowerment (“social/human assets (“assets”), “intrinsic agency” (attitudes about intimate partner violence), and “instrumental agency” (influence in household decision making) influences child nutritional status. The study confirmed positive associations between women’s empowerment with the household decision making and indicators of child non-anthropometric health status. According to their finding, “women’s instrumental agency”, which they measured as participation in household decision making is found more relevant for anemia indicator. Heckert et al. (2019) find that children of empowered women concerning purchasing decisions, health decisions, and the composite empowerment index had a lower prevalence of child wasting by controlling household economic status. Although not all the domains of empowerment were equally significant, Alaofè et al.(2017), reported positive associations between women’s composite empowerment, leadership, and female child's dietary diversity score. Malapit and Quisumbing (2015) found a strong association between women’s empowerment and the quality of infant and young child feeding practices. Ross-Suits (2010) found that maternal autonomy as measured if the woman had the final say in the decision regarding her own healthcare, was statistically significantly associated with child nutritional outcome after controlling for demographic variables. A similar study by Brunson et al. (2009) found that greater levels of women’s autonomy were significantly associated with improved nutrition among older age 3–10 years children. Na et al. (2015) found that the recommended infant and young child feeding (IYCF) criteria were positively associated with the economic dimensions of women empowerment. According to the latter study, the composite empowerment of women was consistently and positively associated with multiple IYCF practices in Mali, Rwanda and Sierra Leone.

On the other hand, some of the studies included in the review did not present the significant association between different domains of women’s empowerment and anthropometric child health outcome (Zereyesus et al. 2017; Deutsch and Silber 2017). Using data from the 2012 population-based survey conducted in Ghana, Zereyesus et al. (2017) found that both the composite empowerment score used to capture women’s empowerment in agriculture and its decomposed components were not statistically significant in their associations with the latent children’s health status (anthropometric indicators of height-for-age and weight-for-height z-scores). The authors noted that their results pertain specifically if the empowerment domains used to construct in the context of farming families matter to children’s health status in northern Ghana. Similarly, Deutsch and Silber (2017) assessed whether women’s empowerment affects the health of children using data from the 2009 Mozambique DHS. Using children's height and weight as the measure of child health outcome, the study did not find an impact of women’s empowerment on the health of children, although they showed children’s health is better when the woman opposes her partner’s violence. This study found that only empowerment domains that have a significant positive influence on the health of children are maternal wealth and the fact that the women do not justify being beating by her husband/partner.

In terms of children’s gender, some studies demonstrated that female children are less likely to be stunted or wasted as compared to male children. For instance, in Tanzania Ross-Suits (2010) found that female children had decreased the odds of being underweight. The reason for this according to different studies might be either female children particularly in sub-Saharan Africa are better nourished than male ones or male children are biologically at greater risk of stunting (Ross-Suits 2010). Women’s empowerment has also a differential impact on male and female children’s health status. Deutsch and Silber (2017) found that the health of female children is better when the woman opposes domestic violence and when her education level is higher. The study by Alaofè et al. (2017) also found the empowerment dimension of mobility (women’s freedom to leave the home) was positively associated with female children’s Weight-for-Height Z score (WHZ) and male children’s Height-for-Age Z score (HAZ) and Weight-for-Age Z score (WAZ), while decision-making was associated with female children’s WAZ and with male child’s WHZ.

#### Women’s Empowerment and Children’s Non-anthropometric Health Indicators

Four out of 15 studies considered infant mortality as a child health outcome (Akinyemi et al. 2017; Alemayehu et al. 2015; Desai and Johnson 2005; Fantahun et al. 2007). According to Fantahun et al. (2007), low decision-making capacity of women and low social capital scores associated with high under-five mortality in Ethiopia. Similarly, Alemayehu et al. (2015) also reported female education and empowerment were inversely associated with infant death. Another study in Nigeria reports that childhood mortality at 59 months is higher among children of women with a low decision-making index compared to those children of women with high decision-making index (Akinyemi et al. 2017). Childhood immunization outcomes are also considered as a measure of children’s health outcomes (Ibrahim et al. 2015). Ibrahim et al.(2015) found a strong and positive influence of the active participation of women in making decisions in the household on their children’s health status measured by their immunization status and nutritional status.

Using data from the 2016 Zimbabwe DHS, Ndaimani et al. (2018) found no statistically significant association between women’s empowerment and childhood vaccinations and diarrhea treatment after controlling for sociodemographic factors. However, this study finds that covariates including being in the middle wealth quintile compared with the poorest wealth quintile, having visited a health facility in the past 12 months, and having health insurance were more likely to predict access to basic vaccines. Desai and Johnson (2005) examined the impact of women’s ability to make independent decisions on children’s health outcomes—particularly vaccination status, nutritional status, and child mortality in 12 developing countries including Benin, Malawi, Mali, Uganda, and Zimbabwe. The result of this study showed that women's decision-making authority had a weak effect on children’s health status in these sub-Saharan African countries.

## Discussion and Conclusions

This systematic review identified 15 studies that examined the association between women’s empowerment and children’s health status in sub-Saharan African countries. Although the methods and the indicators used to measure women’s empowerment vary across the studies, the review finds evidence that women’s empowerment at the family level is positively associated with different children’s health outcomes in most of the sub-Saharan African countries. Also, this review indicates different domains of women’s empowerment may relate differently to different children’s health and nutritional outcomes and that the strength and direction of the association may vary with the children’s and women’s socio-economic characteristics.

This systematic review revealed that most studies used the indicator of women’s autonomy, agency, and decision-making power to examine the association between women’s empowerment and children’s health. The results indicate that, in sub-Saharan African countries, higher decision-making power of women is positively associated with better children’s health status, although some studies showed no association between them. Besides, the majority of the studies used children's nutritional statuses of height-for-age, weight-for-height, and weight-for-age as indicators of children’s health status. Other indicators of health status such as infectious diseases remain limited.

This review underlines some important limitations of the literature included in this systematic review. First, except for Heckert et al. (2019), all the studies included in this systematic review used cross-sectional data, which makes it difficult to stipulate the direction of the relationships identified and/or the direction of causality. All the associations reported lack a temporal direction relationship. In most of the studies, women’s empowerment is measured with respect to the date of the interview of the women; however, information on some of the children’s health status was measured some weeks or months before the interview. For example, in most of the DHS data information regarding the treatment of diarrhea is taken 2 weeks before the survey. Future studies may require more longitudinal and randomized trials to establish appropriate time ordering and to test the causal impact of women’s empowerment on a specific children’s health outcome.

Second, the majority of the included studies used DHS data, indicating heavy positive use of the DHS data as a source for information on women’s empowerment. However, studies that use DHS data are also limited by data availability in terms of the number of empowerment domains and children’s health indicators that could be used for empirical analyses. Given the multidimensionality of empowerment, future studies may benefit by collecting primary data into a specific context to incorporate comprehensive domains of women’s empowerment as in Alkire et al. (2013) recently developed Women’s Empowerment in Agriculture Index (WEAI). Context-specific measures of empowerment have the advantage of capturing locally relevant domains of empowerment. Besides, future studies would benefit by incorporating additional aspects of women's empowerment, such as psychological, political representations and legal, intrinsic agency, mobility, and collective agency (Heckert et al. 2019). Qualitative research may also provide a more in-depth understanding of the contexts connecting different associations between women’s empowerment measures and children’s health outcomes (Alaofè et al. 2017). Studies suggested that conducting qualitative research may help to include more context-specific and validated measures of empowerment (Alaofè et al. 2017; Carlson et al. 2015; Upadhyay and Karasek 2012). For instance, Alaofè et al. (2017) suggested qualitative research could help to include and understand specific contexts related to empowerment measures in the area of women’s participation in labor and political groups by identifying appropriate indicators for these measures. The qualitative method involves the development of scale items that capture women’s empowerment in a variety of contexts, testing those items and conducting psychometric analyses to ensure reliability and validity—especially construct validity (Upadhyay and Karasek 2012).

This review contributes to the existing literature by consolidating the existing evidence regarding the impact of women's empowerment on children's health outcomes in sub-Saharan African countries. Women’s empowerment is now widely accepted as a critical pathway in and of itself to achieving development objectives and contributes to better family health outcomes including child health (Duflo 2012; Mehra 1997). However, women's empowerment in developing countries, particularly in South Asia and sub-Saharan African countries, remains low compared to other regions in the world (Carlson et al. 2015; Cunningham et al. 2015; Smith et al. 2003). This low status of women is a barrier to development and human capital formation, and this might contribute to poor child health and growth. Therefore, the empowerment of women should be diligently sought as a policy tool in the study region to help improve the well-being of the women and the children under their care. This could be done by promoting equal access to education, health, and control over economically significant resources and benefits to promote the welfare, development, and protection of women and their children.

## Electronic supplementary material

Below is the link to the electronic supplementary material.

Electronic supplementary material 1 (DOCX 22 kb)
