# Supplementary material for: Women’s Empowerment and Infant and Child Health Status in Sub-Saharan Africa: A Systematic Review
Source: Matern Child Health J. 2020 Nov 23;25(1):95–106. doi: 10.1007/s10995-020-03025-y (PMC7822794; doi:10.1007/s10995-020-03025-y)
Supplement: Supplementary file 1 — Electronic supplementary material 1 (DOCX 22 kb) [file 10995_2020_3025_MOESM1_ESM.docx]

**Women’s Empowerment and Infant and Children’s Health Status in sub-Saharan Africa. A Systematic Review**

**Additional Materials**

**Appendix A**: Search strings used for systematic search of women’s empowerment and infant and child health status in sub-Saharan Africa. A systematic review

| **Database** | **Year** | **Search strategy** | **Reference identified** |
| --- | --- | --- | --- |
| PubMed | 2019 | (((Women's empowerment) OR female empowerment) OR mother) AND child health) OR children's health) OR Infant health) OR stunting) OR wasting) OR underweight) OR anemia) OR Pneumonia) AND “Sub-Saharan African countries””))) | 1181 |
| Embase | 2019 | (women’s empowerment* OR female empowerment* AND "child health*" OR "Children’s health*") | 690 |
| Google scholar | 2019 | Women’s Empowerment and Infant OR Children’s Health Status | 120 |
| Web of science | 2019 | Women’s Empowerment and Infant OR Child Health Status | 1479 |
| Scopus | 2019 | Women's Empowerment AND Infant OR Child Health Status AND sub-Saharan Africa OR Africa | 1248 |
| **Total number of citations identified** | | | 4,718 |

**Appendix B:** Patient Intervention Comparator Outcome (PICO) strategy.

| **Criteria** |  |
| --- | --- |
| Population | Women age 15-49 years who have under age 5 children who live with them. |
| Intervention | Children’s health outcomes (Anthropometric and non-anthropometric) |
| Comparators | Any treatment for stated Interventions |
| Outcomes | Improved children’s health |
| Timepoints/ follow-up | Any |
| Study type | Cross-sectional and Comparative studies |
| Publication date | 2000– 2019 |
| Publication language | English |
| Setting | Sub-Saharan African countries |

**Appendix C:** Critical assessment criteria of the studies

Studies that meet the eligibility criteria were subject to quality appraisal. The risk of bias is assessed in terms of study design, confounding, selection bias and further statistical issues. Only studies that meet these quality criteria were eventually included in the review. Studies that demonstrated higher quality (rating “A” or “B”) were included in the final review. Studies that scored “C” were excluded from the review.

| **Criteria** | **Items** | **Quality rating** |
| --- | --- | --- |
| Study design and data collection | Data collection based on piloting or pre-testing of tool; OR checks on validity of data; OR tool shown to be reliable in relevant population. | A |
|  | Data collection tool based on previous research, but no piloting or checking, and reliability not demonstrated. | B |
|  | Data collection unclear; OR tools not piloted, checked or based on previous research. | C |
| Confounding variables | Studies adjusted for key variables (SES, age, ethnicity) in the analysis and provided supporting data based on previous research. | A |
|  | Stated that they adjusted for key variables (SES, age, ethnicity) in the analysis but failed to provide supporting data. | B |
|  | No adjustment for key variables reported. | C |
| Selection bias | Selected study sample very likely to represent population from target area AND 80 to 100% response at baseline. | A |
|  | Selected study sample very likely to represent population from target area AND 60 to 79% response at baseline. | B |
|  | Selected study sample very likely to represent population from target area <60% baseline response; OR Somewhat likely to represent population AND <80% response; OR  Not likely to represent population OR representativeness NR/unclear; OR  Response rate at baseline NR/unclear | C |
